# Supplementary material for: Pulse pressure modifies the association between diastolic blood pressure and decrease in kidney function: the Japan Specific Health Checkups Study
Source: Clin Kidney J. 2024 May 25;17(6):sfae152. doi: 10.1093/ckj/sfae152 (PMC11153873; doi:10.1093/ckj/sfae152)
Supplement: sfae152_Supplemental_Files [file sfae152_supplemental_files.zip › 1309 suppl.docx]

**Pulse pressure modifies the association between diastolic blood pressure and decrease in kidney function: The Japan Specific Health Checkups Study**

Hiroyuki Tamaki, MD^1^, Masahiro Eriguchi, MD, PhD^1^, Hisako Yoshida, PhD^2^, Takayuki Uemura, MD^1^, Hikari Tasaki, MD^1^, Masatoshi Nishimoto, MD, PhD^1^, Takaaki Kosugi, MD, PhD^1^, Ken-ichi Samejima, MD, PhD^1^, Kunitoshi Iseki, MD, PhD^3^, Shouichi Fujimoto, MD, PhD^3^, Tsuneo Konta, MD, PhD^3^, Toshiki Moriyama, MD, PhD^3^, Kunihiro Yamagata, MD, PhD^3^, Ichiei Narita, MD, PhD^3^, Masato Kasahara, MD, PhD^3^, Yugo Shibagaki, MD, PhD^3^, Masahide Kondo, MD, PhD^3^, Koichi Asahi, MD, PhD^3^, Tsuyoshi Watanabe, MD, PhD^3^, Kazuhiko Tsuruya, MD, PhD^1,3^

^1^Department of Nephrology, Nara Medical University, Kashihara, Nara, Japan

^2^Department of Medical Statistics, Osaka Metropolitan University Graduate School of Medicine, Osaka, Osaka, Japan

^3^Steering Committee of The Japan Specific Health Checkups (J‑SHC) Study, Fukushima, Japan

**Corresponding Author:** Masahiro Eriguchi, MD, PhD

Department of Nephrology, Nara Medical University

840 Shijo-cho, Kashihara, Nara 634-8521, Japan

Phone: +81-744-29-8865; Fax: +81-744-23-9913

E-mail: meriguci@gmail.com

**Supplemental data**

# **Supplemental Table S1**. Baseline characteristics according to diastolic blood pressure in participants with low pulse pressure (≤39 mmHg).

|  | | **DBP, mmHg** | | | | | |
| --- | --- | --- | --- | --- | --- | --- | --- |
|  |  | **≤60** | **61–80** | **81–100** | **≥101** | **SMD** | **Missing, %** |
| n | | 10086 | 57136 | 22758 | 913 |  |  |
| Age, years | | 56.8 (10.0) | 58.6 (9.2) | 59.4 (8.6) | 58.0 (8.9) | 0.15 | 0 |
| Sex, female, n (%) | | 7702 (76.4) | 34619 (60.6) | 9743 (42.8) | 255 (27.9) | 0.59 | 0 |
| BMI, kg/m^2^ | | 20.7 (2.7) | 22.1 (3.1) | 23.7 (3.4) | 24.9 (3.8) | 0.73 | 0.6 |
| SBP, mmHg | | 91.6 (4.9) | 105.8 (6.5) | 121.3 (6.3) | 140.0 (7.0) | 4.34 | 0 |
| DBP, mmHg | | 57.2 (3.5) | 71.6 (5.5) | 87.2 (4.9) | 106.8 (6.0) | 5.52 | 0 |
| Pulse pressure, mmHg | | 34.4 (3.7) | 34.2 (4.0) | 34.2 (4.4) | 33.1 (5.4) | 0.14 | 0 |
| Antihypertensive agents, n (%) | | 326 (3.3) | 5573 (10.0) | 5817 (26.1) | 277 (31.3) | 0.47 | 2.3 |
| Antidiabetic agents, n (%) | | 237 (2.4) | 1405 (2.5) | 646 (2.9) | 29 (3.3) | 0.03 | 2.3 |
| Cardiovascular disease, n (%) | | 378 (4.2) | 2278 (4.5) | 1067 (5.2) | 46 (5.6) | 0.04 | 10.9 |
| Stroke, n (%) | | 141 (1.6) | 1198 (2.4) | 703 (3.4) | 41 (5.0) | 0.11 | 11 |
| Current smoking, n (%) | | 1834 (18.6) | 10138 (18.2) | 4250 (19.1) | 188 (21.2) | 0.04 | 2.5 |
| HbA1c, % | | 5.2 (0.6) | 5.2 (0.6) | 5.3 (0.7) | 5.3 (0.8) | 0.13 | 2 |
| Serum creatinine, mg/dL | | 0.7 (0.2) | 0.7 (0.2) | 0.8 (0.2) | 0.8 (0.2) | 0.38 | 0 |
| eGFR, mL/min/1.73 m^2^ | | 77.8 (15.8) | 76.5 (15.3) | 75.2 (15.5) | 74.4 (15.8) | 0.12 | 0 |
| Proteinuria, n (%) | (-) | 9019 (89.7) | 51198 (89.9) | 19598 (86.3) | 724 (79.5) | 0.20 | 0.3 |
|  | (±) | 744 (7.4) | 4095 (7.2) | 1876 (8.3) | 82 (9.0) |  |  |
|  | (1+) | 219 (2.2) | 1311 (2.3) | 897 (3.9) | 75 (8.2) |  |  |
|  | (2+) | 55 (0.5) | 290 (0.5) | 266 (1.2) | 27 (3.0) |  |  |
|  | (3+) | 14 (0.1) | 75 (0.1) | 75 (0.3) | 3 (0.3) |  |  |

Data are presented as median (interquartile range) or number (%). SMD, standardized mean difference

BMI, body mass index; DBP, diastolic blood pressure; SBP, systolic blood pressure; HbA1c, hemoglobin A1c; eGFR, estimated glomerular filtration rate

# **Supplemental Table S2**. Baseline characteristics according to diastolic blood pressure in participants with normal pulse pressure (40–59 mmHg).

|  | | **DBP, mmHg** | | | | | |
| --- | --- | --- | --- | --- | --- | --- | --- |
|  |  | **≤60** | **61–80** | **81–100** | **≥101** | **SMD** | **Missing, %** |
| n | | 35258 | 266558 | 124292 | 4811 |  |  |
| Age, years | | 59.9 (9.7) | 62.3 (8.2) | 62.4 (7.6) | 59.5 (8.4) | 0.22 | 0 |
| Sex, female, n (%) | | 25727 (73.0) | 162395 (60.9) | 59865 (48.2) | 1599 (33.2) | 0.47 | 0 |
| BMI, kg/m^2^ | | 21.7 (2.9) | 23.0 (3.2) | 24.0 (3.4) | 24.9 (3.8) | 0.54 | 0.8 |
| SBP, mmHg | | 105.1 (6.6) | 122.0 (7.8) | 136.9 (7.2) | 156.7 (7.5) | 3.9 | 0 |
| DBP, mmHg | | 57.2 (3.8) | 72.9 (5.4) | 87.3 (4.8) | 106.5 (5.3) | 5.65 | 0 |
| Pulse pressure, mmHg | | 47.9 (5.5) | 49.1 (5.5) | 49.5 (5.4) | 50.2 (5.6) | 0.22 | 0 |
| Antihypertensive agents, n (%) | | 3293 (9.5) | 61845 (23.6) | 44371 (36.3) | 1437 (30.5) | 0.36 | 1.7 |
| Antidiabetic agents, n (%) | | 1418 (4.1) | 12226 (4.7) | 5014 (4.1) | 109 (2.3) | 0.06 | 1.7 |
| Cardiovascular disease, n (%) | | 1713 (5.4) | 14093 (5.9) | 6127 (5.5) | 182 (4.3) | 0.04 | 10.6 |
| Stroke, n (%) | | 752 (2.4) | 7731 (3.2) | 4259 (3.9) | 135 (3.2) | 0.04 | 10.7 |
| Current smoking, n (%) | | 5397 (15.6) | 38123 (14.6) | 19394 (15.9) | 1045 (22.3) | 0.1 | 1.9 |
| HbA1c, % | | 5.3 (0.6) | 5.3 (0.6) | 5.4 (0.7) | 5.4 (0.8) | 0.09 | 2.1 |
| Serum creatinine, mg/dL | | 0.7 (0.2) | 0.7 (0.2) | 0.7 (0.2) | 0.8 (0.2) | 0.28 | 0 |
| eGFR, mL/min/1.73 m^2^ | | 77.3 (15.9) | 75.8 (15.6) | 75.1 (15.5) | 75.4 (15.9) | 0.07 | 0 |
| Proteinuria, n (%) | (-) | 31727 (90.2) | 236205 (88.8) | 106397 (85.8) | 3766 (78.5) | 0.22 | 0.2 |
|  | (±) | 2491 (7.1) | 19447 (7.3) | 10247 (8.3) | 457 (9.5) |  |  |
|  | (1+) | 733 (2.1) | 7721 (2.9) | 5273 (4.3) | 349 (7.3) |  |  |
|  | (2+) | 175 (0.5) | 2076 (0.8) | 1697 (1.4) | 178 (3.7) |  |  |
|  | (3+) | 42 (0.1) | 546 (0.2) | 427 (0.3) | 49 (1.0) |  |  |

Data are presented as median (interquartile range) or number (%). SMD, standardized mean difference

BMI, body mass index; DBP, diastolic blood pressure; SBP, systolic blood pressure; HbA1c, hemoglobin A1c; eGFR, estimated glomerular filtration rate

|  | | **DBP, mmHg** | | | | | |
| --- | --- | --- | --- | --- | --- | --- | --- |
|  |  | **≤60** | **61–80** | **81–100** | **≥101** | **SMD** | **Missing, %** |
| n | | 15225 | 116309 | 66195 | 5481 |  |  |
| Age, years | | 65.6 (6.9) | 66.2 (5.8) | 65.1 (6.0) | 62.3 (7.3) | 0.3 | 0 |
| Sex, female, n (%) | | 10205 (67.0) | 71667 (61.6) | 35107 (53.0) | 2291 (41.8) | 0.29 | 0 |
| BMI, kg/m^2^ | | 22.8 (3.2) | 23.6 (10.0) | 24.1 (3.4) | 24.7 (3.7) | 0.24 | 0.8 |
| SBP, mmHg | | 126.7 (10.2) | 140.7 (9.8) | 157.4 (10.6) | 181.2 (14.3) | 2.59 | 0 |
| DBP, mmHg | | 57.3 (4.4) | 73.1 (5.4) | 88.4 (5.2) | 107.8 (6.7) | 5.08 | 0 |
| Pulse pressure, mmHg | | 69.4 (10.2) | 67.7 (8.4) | 69.0 (9.0) | 73.4 (11.8) | 0.29 | 0 |
| Antihypertensive agents, n (%) | | 4991 (33.2) | 50019 (43.5) | 28359 (43.5) | 1501 (27.9) | 0.2 | 1.3 |
| Antidiabetic agents, n (%) | | 1600 (10.6) | 10125 (8.8) | 4038 (6.2) | 142 (2.6) | 0.18 | 1.5 |
| Cardiovascular disease, n (%) | | 1253 (9.0) | 8050 (7.6) | 3449 (5.9) | 176 (3.7) | 0.12 | 9.9 |
| Stroke, n (%) | | 593 (4.3) | 4912 (4.7) | 2434 (4.1) | 153 (3.2) | 0.04 | 9.9 |
| Current smoking, n (%) | | 1973 (13.1) | 14436 (12.6) | 9449 (14.6) | 1161 (21.6) | 0.13 | 1.6 |
| HbA1c, % | | 5.5 (0.7) | 5.5 (0.7) | 5.4 (0.8) | 5.4 (0.9) | 0.01 | 2 |
| Serum creatinine, mg/dL | | 0.7 (0.3) | 0.7 (0.2) | 0.7 (0.2) | 0.8 (0.2) | 0.14 | 0 |
| eGFR, mL/min/1.73 m^2^ | | 74.9 (16.8) | 74.7 (16.1) | 74.7 (15.8) | 74.6 (16.1) | 0.01 | 0 |
| Proteinuria, n (%) | (-) | 13206 (86.9) | 99524 (85.7) | 54910 (83.1) | 4108 (75.1) | 0.18 | 0.2 |
|  | (±) | 1193 (7.8) | 9291 (8.0) | 5760 (8.7) | 613 (11.2) |  |  |
|  | (1+) | 536 (3.5) | 4868 (4.2) | 3516 (5.3) | 451 (8.2) |  |  |
|  | (2+) | 209 (1.4) | 1795 (1.5) | 1428 (2.2) | 233 (4.3) |  |  |
|  | (3+) | 56 (0.4) | 631 (0.5) | 459 (0.7) | 66 (1.2) |  |  |

# **Supplemental Table S3**. Baseline characteristics according to diastolic blood pressure in participants with high pulse pressure (≥60 mmHg).

Data are presented as median (interquartile range) or number (%). SMD, standardized mean difference

BMI, body mass index; DBP, diastolic blood pressure; SBP, systolic blood pressure; HbA1c, hemoglobin A1c; eGFR, estimated glomerular filtration rate

# **Supplemental Table S4**. Baseline characteristics according to systolic blood pressure in participants with low pulse pressure (≤39 mmHg).

|  | | **SBP, mmHg** | | | | | |
| --- | --- | --- | --- | --- | --- | --- | --- |
|  |  | **≤100** | **101–130** | **131–160** | **≥161** | **SMD** | **Missing, %** |
| n | | 24035 | 64342 | 2516 | N/A |  |  |
| Age, years | | 57.2 (9.7) | 59.1 (9.0) | 58.6 (8.6) | N/A | 0.14 | 0 |
| Sex, female, n (%) | | 17298 (72.0) | 34213 (53.2) | 808 (32.1) | N/A | 0.57 | 0 |
| BMI, kg/m^2^ | | 21.1 (2.8) | 22.8 (3.2) | 24.6 (3.6) | N/A | 0.73 | 0.6 |
| SBP, mmHg | | 94.9 (4.9) | 112.5 (7.4) | 136.1 (5.2) | N/A | 4.88 | 0 |
| DBP, mmHg | | 62.4 (5.9) | 77.7 (7.7) | 100.1 (6.4) | N/A | 3.85 | 0 |
| Pulse pressure, mmHg | | 32.4 (4.7) | 34.8 (3.7) | 36.0 (3.3) | N/A | 0.6 | 0 |
| Antihypertensive agents, n (%) | | 1011 (4.3) | 10190 (16.2) | 792 (32.3) | N/A | 0.52 | 2.3 |
| Antidiabetic agents, n (%) | | 532 (2.3) | 1715 (2.7) | 70 (2.9) | N/A | 0.03 | 2.3 |
| Cardiovascular disease, n (%) | | 884 (4.1) | 2759 (4.8) | 126 (5.6) | N/A | 0.05 | 10.9 |
| Stroke, n (%) | | 383 (1.8) | 1597 (2.8) | 103 (4.6) | N/A | 0.11 | 11 |
| Current smoking, n (%) | | 4239 (18.1) | 11645 (18.6) | 526 (21.5) | N/A | 0.06 | 2.5 |
| HbA1c, % | | 5.2 (0.6) | 5.3 (0.6) | 5.3 (0.7) | N/A | 0.13 | 2 |
| Serum creatinine, mg/dL | | 0.7 (0.2) | 0.7 (0.2) | 0.8 (0.2) | N/A | 0.35 | 0 |
| eGFR, mL/min/1.73 m^2^ | | 77.4 (15.6) | 76.0 (15.3) | 74.6 (16.2) | N/A | 0.12 | 0 |
| Proteinuria, n (%) | (-) | 21511 (89.8) | 56954 (88.7) | 2074 (82.6) | N/A | 0.18 | 0.3 |
|  | (±) | 1778 (7.4) | 4805 (7.5) | 214 (8.5) | N/A |  |  |
|  | (1+) | 523 (2.2) | 1826 (2.8) | 153 (6.1) | N/A |  |  |
|  | (2+) | 111 (0.5) | 469 (0.7) | 58 (2.3) | N/A |  |  |
|  | (3+) | 31 (0.1) | 125 (0.2) | 11 (0.4) | N/A |  |  |

Data are presented as median (interquartile range) or number (%). SMD, standardized mean difference

BMI, body mass index; DBP, diastolic blood pressure; SBP, systolic blood pressure; HbA1c, hemoglobin A1c; eGFR, estimated glomerular filtration rate

# **Supplemental Table S5**. Baseline characteristics according to systolic blood pressure in participants with normal pulse pressure (40–59 mmHg).

|  | | **SBP, mmHg** | | | | | |
| --- | --- | --- | --- | --- | --- | --- | --- |
|  |  | **≤100** | **101–130** | **131–160** | **≥161** | **SMD** | **Missing, %** |
| n | | 10377 | 286636 | 132650 | 1256 |  |  |
| Age, years | | 57.8 (10.0) | 61.8 (8.4) | 63.1 (7.3) | 59.3 (8.6) | 0.36 | 0 |
| Sex, female, n (%) | | 7990 (77.0) | 174377 (60.8) | 66807 (50.4) | 412 (32.8) | 0.51 | 0 |
| BMI, kg/m^2^ | | 21.2 (2.8) | 22.9 (3.2) | 24.0 (3.4) | 25.3 (4.0) | 0.66 | 0.8 |
| SBP, mmHg | | 97.3 (3.5) | 120.0 (7.5) | 138.6 (6.1) | 166.0 (5.1) | 7.12 | 0 |
| DBP, mmHg | | 54.7 (4.8) | 72.1 (7.4) | 86.2 (6.9) | 111.2 (6.4) | 4.92 | 0 |
| Pulse pressure, mmHg | | 42.7 (3.3) | 47.8 (5.2) | 52.4 (4.6) | 54.7 (3.9) | 1.66 | 0 |
| Antihypertensive agents, n (%) | | 484 (4.7) | 60632 (21.5) | 49490 (37.9) | 340 (27.9) | 0.46 | 1.7 |
| Antidiabetic agents, n (%) | | 289 (2.8) | 12485 (4.4) | 5966 (4.6) | 27 (2.2) | 0.08 | 1.7 |
| Cardiovascular disease, n (%) | | 415 (4.4) | 14872 (5.8) | 6789 (5.8) | 39 (3.6) | 0.06 | 10.6 |
| Stroke, n (%) | | 154 (1.6) | 8100 (3.2) | 4589 (3.9) | 34 (3.1) | 0.07 | 10.7 |
| Current smoking, n (%) | | 1672 (16.4) | 42279 (15.0) | 19707 (15.1) | 301 (24.7) | 0.13 | 1.9 |
| HbA1c, % | | 5.2 (0.5) | 5.3 (0.6) | 5.4 (0.7) | 5.4 (0.9) | 0.17 | 2.1 |
| Serum creatinine, mg/dL | | 0.7 (0.2) | 0.7 (0.2) | 0.7 (0.2) | 0.8 (0.2) | 0.3 | 0 |
| eGFR, mL/min/1.73 m^2^ | | 78.0 (15.9) | 76.0 (15.6) | 75.1 (15.6) | 75.4 (16.6) | 0.1 | 0 |
| Proteinuria, n (%) | (-) | 9307 (89.9) | 254482 (89.0) | 113355 (85.6) | 951 (75.9) | 0.26 | 0.2 |
|  | (±) | 777 (7.5) | 20792 (7.3) | 10954 (8.3) | 119 (9.5) |  |  |
|  | (1+) | 210 (2.0) | 8074 (2.8) | 5690 (4.3) | 102 (8.1) |  |  |
|  | (2+) | 49 (0.5) | 2112 (0.7) | 1904 (1.4) | 61 (4.9) |  |  |
|  | (3+) | 6 (0.1) | 539 (0.2) | 499 (0.4) | 20 (1.6) |  |  |

Data are presented as median (interquartile range) or number (%). SMD, standardized mean difference

BMI, body mass index; DBP, diastolic blood pressure; SBP, systolic blood pressure; HbA1c, hemoglobin A1c; eGFR, estimated glomerular filtration rate

# **Supplemental Table S6**. Baseline characteristics according to systolic blood pressure in participants with high pulse pressure (≥60 mmHg).

|  | | **SBP, mmHg** | | | | | |
| --- | --- | --- | --- | --- | --- | --- | --- |
|  |  | **≤100** | **101–130** | **131–160** | **≥161** | **SMD** | **Missing, %** |
| n | | N/A | 29630 | 144023 | 29557 |  |  |
| Age, years | | N/A | 65.2 (6.9) | 65.9 (5.8) | 65.2 (6.1) | 0.08 | 0 |
| Sex, female, n (%) | | N/A | 19502 (65.8) | 84532 (58.7) | 15236 (51.5) | 0.19 | 0 |
| BMI, kg/m^2^ | | N/A | 22.9 (3.2) | 23.8 (9.1) | 24.3 (3.5) | 0.21 | 0.8 |
| SBP, mmHg | | N/A | 125.8 (4.7) | 145.1 (8.0) | 172.2 (10.9) | 3.76 | 0 |
| DBP, mmHg | | N/A | 63.1 (5.9) | 77.9 (8.3) | 92.2 (11.0) | 2.28 | 0 |
| Pulse pressure, mmHg | | N/A | 62.8 (3.8) | 67.2 (6.7) | 79.9 (11.8) | 1.36 | 0 |
| Antihypertensive agents, n (%) | | N/A | 9019 (30.8) | 63809 (44.9) | 12042 (41.4) | 0.2 | 1.3 |
| Antidiabetic agents, n (%) | | N/A | 2549 (8.7) | 11396 (8.0) | 1960 (6.8) | 0.05 | 1.5 |
| Cardiovascular disease, n (%) | | N/A | 2121 (7.9) | 9297 (7.2) | 1510 (5.8) | 0.06 | 9.9 |
| Stroke, n (%) | | N/A | 1164 (4.3) | 5836 (4.5) | 1092 (4.2) | 0.01 | 9.9 |
| Current smoking, n (%) | | N/A | 3764 (12.9) | 18492 (13.1) | 4763 (16.4) | 0.07 | 1.6 |
| HbA1c, % | | N/A | 5.4 (0.7) | 5.5 (0.7) | 5.5 (0.9) | 0.05 | 2 |
| Serum creatinine, mg/dL | | N/A | 0.7 (0.2) | 0.7 (0.2) | 0.7 (0.2) | 0.11 | 0 |
| eGFR, mL/min/1.73 m^2^ | | N/A | 75.1 (16.2) | 74.7 (16.0) | 74.4 (16.1) | 0.03 | 0 |
| Proteinuria, n (%) | (-) | N/A | 26009 (87.9) | 122247 (85.0) | 23492 (79.7) | 0.18 | 0.2 |
|  | (±) | N/A | 2229 (7.5) | 11852 (8.2) | 2776 (9.4) |  |  |
|  | (1+) | N/A | 963 (3.3) | 6478 (4.5) | 1930 (6.5) |  |  |
|  | (2+) | N/A | 297 (1.0) | 2398 (1.7) | 970 (3.3) |  |  |
|  | (3+) | N/A | 75 (0.3) | 813 (0.6) | 324 (1.1) |  |  |

Data are presented as median (interquartile range) or number (%). SMD, standardized mean difference

BMI, body mass index; DBP, diastolic blood pressure; SBP, systolic blood pressure; HbA1c, hemoglobin A1c; eGFR, estimated glomerular filtration rate.

# **Supplemental Table S7**. Baseline characteristics according to pulse pressure.

|  | | **PP, mmHg** | | | | | |
| --- | --- | --- | --- | --- | --- | --- | --- |
|  |  | **≤39** | **40–59** | **≥60** | **SMD** | **Missing, %** | |
| n | | 90893 | 430919 | 203210 |  | |  |
| Age, years | | 58.6 (9.2) | 62.1 (8.2) | 65.7 (6.0) | 0.6 | | 0 |
| Sex, female, n (%) | | 52319 (57.6) | 249586 (57.9) | 119270 (58.7) | 0.02 | | 0 |
| BMI, kg/m^2^ | | 22.4 (3.2) | 23.2 (3.3) | 23.7 (7.9) | 0.19 | | 0.8 |
| SBP, mmHg | | 108.5 (11.3) | 125.3 (12.1) | 146.2 (15.0) | 1.94 | | 0 |
| DBP, mmHg | | 74.3 (10.8) | 76.2 (10.4) | 77.8 (11.5) | 0.22 | | 0 |
| Pulse pressure, mmHg | | 34.2 (4.1) | 49.1 (5.5) | 68.4 (8.9) | 3.53 | | 0 |
| Antihypertensive agents, n (%) | | 11993 (13.5) | 110946 (26.2) | 84870 (42.3) | 0.45 | | 1.7 |
| Antidiabetic agents, n (%) | | 2317 (2.6) | 18767 (4.4) | 15905 (7.9) | 0.16 | | 1.7 |
| Cardiovascular disease, n (%) | | 3769 (4.7) | 22115 (5.7) | 12928 (7.1) | 0.07 | | 10.4 |
| Stroke, n (%) | | 2083 (2.6) | 12877 (3.3) | 8092 (4.4) | 0.07 | | 10.5 |
| Current smoking, n (%) | | 16410 (18.5) | 63959 (15.1) | 27019 (13.5) | 0.09 | | 1.9 |
| HbA1c, % | | 5.2 (0.6) | 5.3 (0.6) | 5.5 (0.8) | 0.2 | | 2 |
| Serum creatinine, mg/dL | | 0.7 (0.2) | 0.7 (0.2) | 0.7 (0.2) | 0.02 | | 0 |
| eGFR, mL/min/1.73 m^2^ | | 76.3 (15.4) | 75.7 (15.6) | 74.7 (16.0) | 0.07 | | 0 |
| Proteinuria, n (%) | (-) | 80539 (88.9) | 378095 (87.9) | 171748 (84.7) | 0.11 | | 0.2 |
|  | (±) | 6797 (7.5) | 32642 (7.6) | 16857 (8.3) |  |  |  |
|  | (1+) | 2502 (2.8) | 14076 (3.3) | 9371 (4.6) |  |  |  |
|  | (2+) | 638 (0.7) | 4126 (1.0) | 3665 (1.8) |  |  |  |
|  | (3+) | 167 (0.2) | 1064 (0.2) | 1212 (0.6) |  |  |  |

Data are presented as median (interquartile range) or number (%). SMD, standardized mean difference

BMI, body mass index; DBP, diastolic blood pressure; SBP, systolic blood pressure; HbA1c, hemoglobin A1c; eGFR, estimated glomerular filtration rate.

# **Supplemental Table S8**. HRs (95% CIs) for 30% eGFR reduction from baseline according to DBP and SBP in stratified PP groups

|  | | **HR (95% CI) for 30% decrease in eGFR** | | |  | **HR (95% CI) for 30% decrease in eGFR** | | |
| --- | --- | --- | --- | --- | --- | --- | --- | --- |
| **PP (mmHg)** | **DBP (mmHg)** | **Crude** | **Age/sex adjusted** | **Fully adjusted^*^** | **SBP (mmHg)** | **Crude** | **Age/sex adjusted** | **Fully adjusted^*^** |
| **≤39** | **≤60** | 0.79 (0.69–0.91) | 0.83 (0.72–0.95) | 0.96 (0.83–1.12) | **≤100** | 0.78 (0.71–0.86) | 0.81 (0.74–0.89) | 0.93 (0.84–1.03) |
|  | **61–80** | 0.78 (0.73–0.83) | 0.82 (0.77–0.87) | 0.96 (0.90–1.03) | **101–130** | 0.85 (0.81–0.90) | 0.89 (0.84–0.95) | 1.01 (0.95–1.07) |
|  | **81–100** | 0.92 (0.84–1.00) | 0.98 (0.90–1.07) | 1.09 (1.00–1.20) | **131–160** | 1.23 (0.98–1.55) | 1.34 (1.07–1.69) | 1.41 (1.11–1.79) |
|  | **≥101** | 1.04 (0.69–1.56) | 1.16 (0.77–1.74) | 1.21 (0.78–1.88) | **≥161** | N/A | N/A | N/A |
| **40–59** | **≤60** | 0.89 (0.82–0.95) | 0.90 (0.84–0.97) | 0.93 (0.86–1.01) | **≤100** | 0.86 (0.75–0.98) | 0.88 (0.77–1.00) | 0.90 (0.78–1.04) |
|  | **61–80** | 1 (Reference) | 1 (Reference) | 1 (Reference) | **101–130** | 1 (Reference) | 1 (Reference) | 1 (Reference) |
|  | **81–100** | 1.06 (1.02–1.11) | 1.08 (1.04–1.13) | 1.05 (1.01–1.10) | **131–160** | 1.15 (1.11–1.20) | 1.15 (1.11–1.20) | 1.05 (1.01–1.10) |
|  | **≥101** | 1.38 (1.18–1.62) | 1.50 (1.28–1.76) | 1.45 (1.22–1.72) | **≥161** | 1.80 (1.36–2.38) | 1.98 (1.47–2.59) | 1.55 (1.14–2.10) |
| **≥60** | **≤60** | 1.54 (1.41–1.68) | 1.46 (1.34–1.60) | 1.26 (1.15–1.38) | **≤100** | N/A | N/A | N/A |
|  | **61–80** | 1.46 (1.40–1.51) | 1.39 (1.33–1.44) | 1.11 (1.07–1.16) | **101–130** | 1.24 (1.16–1.33) | 1.18 (1.10–1.27) | 1.04 (0.96–1.12) |
|  | **81–100** | 1.51 (1.44–1.58) | 1.48 (1.41–1.55) | 1.24 (1.18–1.31) | **131–160** | 1.51 (1.46–1.57) | 1.45 (1.39–1.50) | 1.15 (1.11–1.20) |
|  | **≥101** | 2.06 (1.81–2.34) | 2.13 (1.88–2.42) | 1.86 (1.62–2.14) | **≥161** | 2.02 (1.90–2.14) | 1.97 (1.85–2.09) | 1.50 (1.41–1.60) |

HR, hazard ratio; CI, confidence interval; eGFR, estimated glomerular filtration rate; PP, pulse pressure; DBP, diastolic blood pressure; SBP, systolic blood pressure

Fully adjusted^*^; adjusted for age, sex, body mass index, history of CVD and stroke, current smoking status, antihypertensive and antidiabetic agents’ usage, HbA1c, eGFR and proteinuria

# Supplemental Figure S1**.** Heat maps of the fully adjusted HRs (95% CIs) for 40% eGFR reduction from baseline level stratified by DBP and PP levels **(A)**, and by SBP and PP levels **(B)**. Green: low risk (HRs<1.0); Yellow: moderately increased risk (1.0**≤**HRs<1.2); Orange: high risk (1.2 **≤** HRs<1.4); Red: very high risk (≥1.4).

HR, hazard ratio; CI, confidence interval; PP, pulse pressure; DBP, diastolic blood pressure; SBP, systolic blood pressure; eGFR, estimated glomerular filtration rate

# Supplemental Figure S2**.** The restricted spline curves showing fully adjusted HRs (solid lines) and 95% CIs (shaped areas) for the relationships between 40% eGFR reduction from baseline and baseline diastolic **(A, B)** and systolic **(C, D)** blood pressures in all participants **(A, C)** and those in each PP category **(B, D)**

HR, hazard ratio; CI, confidence interval; PP, pulse pressure; eGFR, estimated glomerular filtration rate.

# **Supplemental Figure S3.** Relationship of diastolic blood pressure and pulse pressure.

Diastolic blood pressure has no (or very weak positive) association with pulse pressure.　R value 0.11, P value <0.001.

# **Supplemental Figure S4**. Relationship of systolic blood pressure and pulse pressure.

Systolic blood pressure has a very strong positive association with pulse pressure. R value 0.79, P value <0.001.
