# Supplementary material for: Pulse pressure modifies the association between diastolic blood pressure and decrease in kidney function: the Japan Specific Health Checkups Study
Source: Clin Kidney J. 2024 May 25;17(6):sfae152. doi: 10.1093/ckj/sfae152 (PMC11153873; doi:10.1093/ckj/sfae152)
Supplement: sfae152_Supplemental_Files [file sfae152_supplemental_files.zip › Revise_Figure_S1.pdf]

Figure S1

A

| | | HR (95% CI) for 40% decrease in eGFR | | |
|---|---|---|---|---|
| | | Pulse pressure (mmHg) | | |
| | | ≤39 | 40–59 | ≥60 |
| DBP (mmHg) | ≤60 | 0.71 (0.51–0.99) | 0.74 (0.62–0.88) | 1.41 (1.19–1.67) |
| | 61–80 | 1.21 (1.08–1.36) | 1 (Reference) | 1.11 (1.03–1.20) |
| | 81–100 | 1.58 (1.37–1.82) | 1.10 (1.02–1.19) | 1.30 (1.19–1.43) |
| | ≥101 | 1.56 (0.81–3.00) | 1.36 (1.00–1.86) | 1.68 (1.30–2.18) |

B

| | | HR (95% CI) for 40% decrease in eGFR | | |
|---|---|---|---|---|
| | | Pulse pressure (mmHg) | | |
| | | ≤39 | 40–59 | ≥60 |
| SBP (mmHg) | ≤100 | 1.05 (0.88–1.26) | 0.77 (0.56–1.05) | N/A |
| | 101–130 | 1.32 (1.19–1.46) | 1 (Reference) | 1.00 (0.86–1.16) |
| | 131–160 | 1.92 (1.34–2.74) | 1.08 (0.99–1.16) | 1.19 (1.10–1.28) |
| | ≥161 | N/A | 1.30 (0.72–2.35) | 1.55 (1.38–1.74) |
