# Supplementary figures and images for: Pulse pressure modifies the association between diastolic blood pressure and decrease in kidney function: the Japan Specific Health Checkups Study
Source: Clin Kidney J. 2024 May 25;17(6):sfae152. doi: 10.1093/ckj/sfae152 (PMC11153873; doi:10.1093/ckj/sfae152)

Figure S2

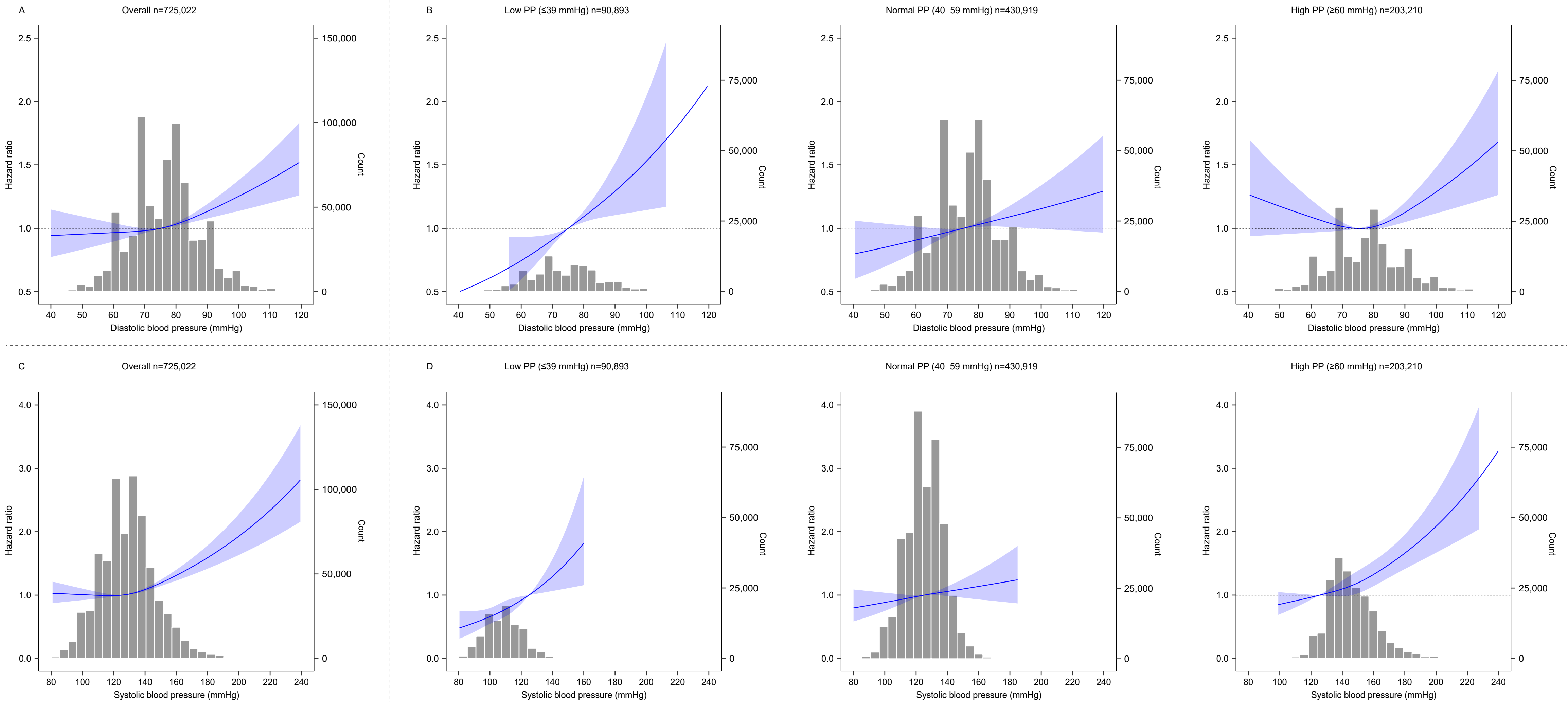

Supplement: sfae152_Supplemental_Files [file sfae152_supplemental_files.zip › Revise_Figure_S2.pdf]

Figure S3

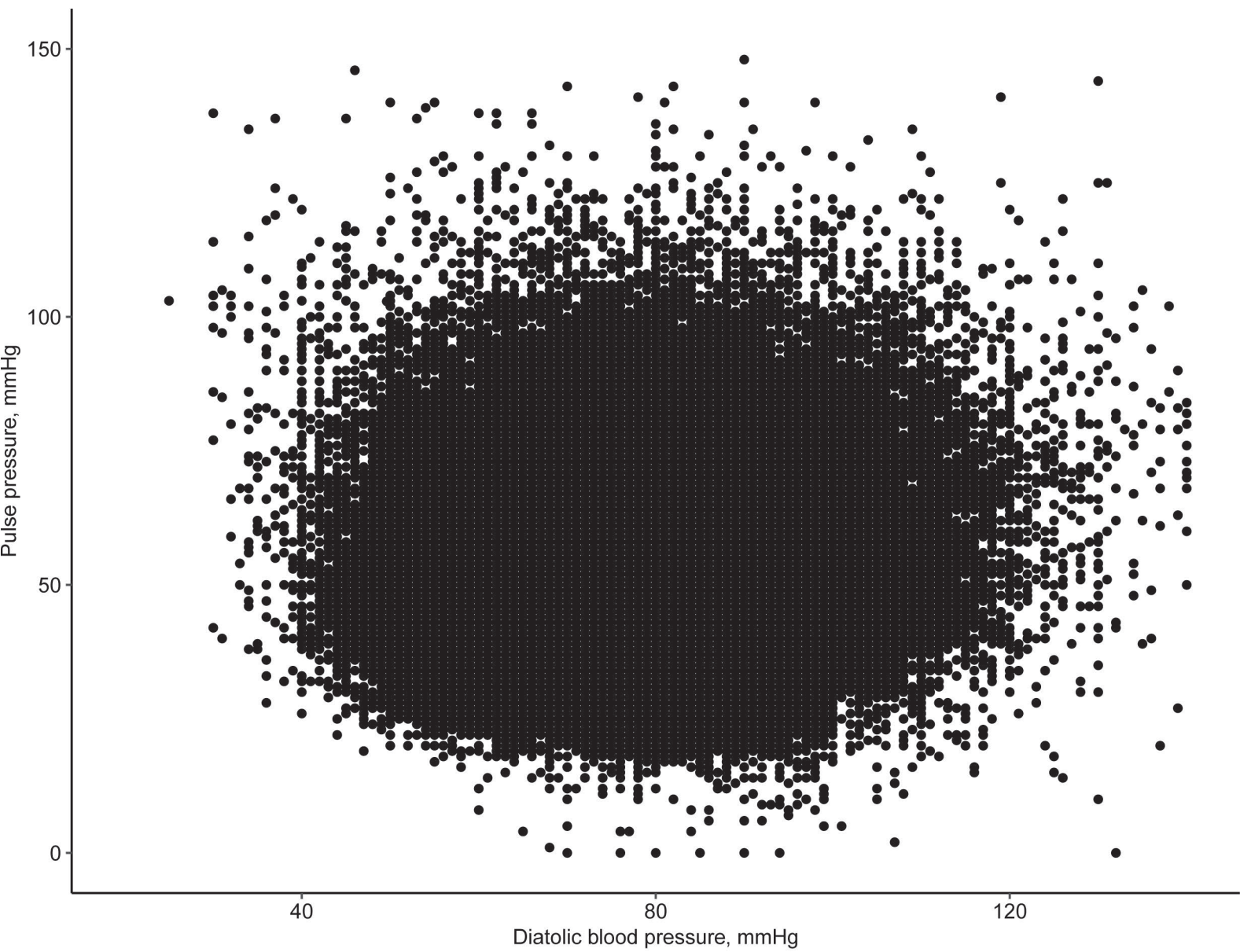

Supplement: sfae152_Supplemental_Files [file sfae152_supplemental_files.zip › Revise_Figure_S3.pdf]

Figure S4

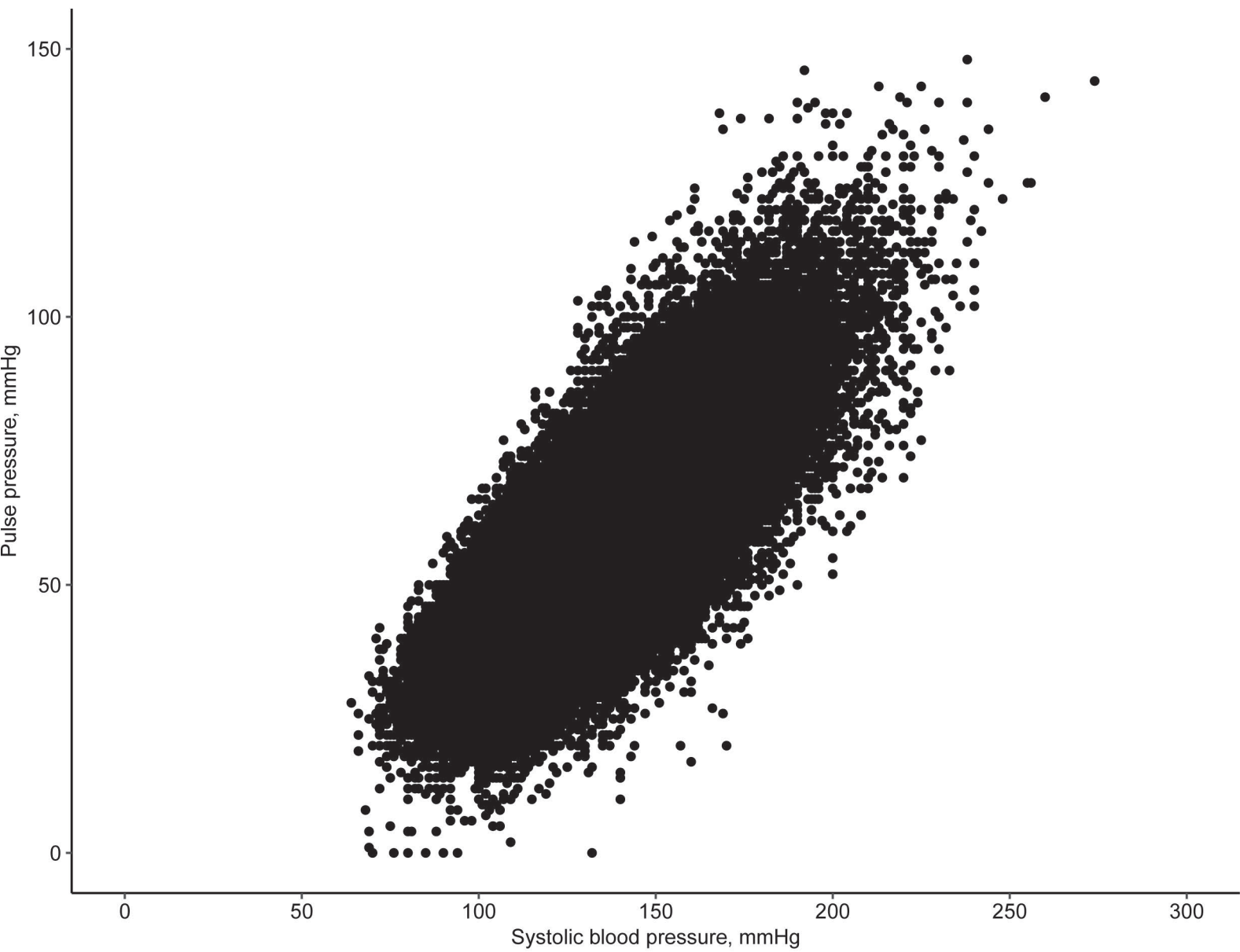

Supplement: sfae152_Supplemental_Files [file sfae152_supplemental_files.zip › Revise_Figure_S4.pdf]
